# FPGA-Based Implementation of Stochastic Configuration Networks for Regression Prediction

**DOI:** 10.3390/s20154191

**Published:** 2020-07-28

**Authors:** Yunqi Gao, Feng Luan, Jiaqi Pan, Xu Li, Yaodong He

**Affiliations:** 1 School of Computer Science and Engineering, Northeastern University, Shenyang 110819, China; 20174852@stu.neu.edu.cn (Y.G.); 20174839@stu.neu.edu.cn (J.P.); 2 Key Laboratory of Intelligent Computing in Medical Image, Ministry of Education, Northeastern University, Shenyang 110819, China; 3 The State Key Laboratory of Rolling and Automation, Northeastern University, Shenyang 110819, China; lixu@ral.neu.edu.cn (X.L.); 1870306@stu.neu.edu.cn (Y.H.)

**Keywords:** field programmable gate array, hardware neural networks, regression prediction, stochastic configuration networks

## Abstract

The implementation of neural network regression prediction based on digital circuits is one of the challenging problems in the field of machine learning and cognitive recognition, and it is also an effective way to relieve the pressure of the Internet in the era of intelligence. As a nonlinear network, the stochastic configuration network (SCN) is considered to be an effective method for regression prediction due to its good performance in learning and generalization. Therefore, in this paper, we adapt the SCN to regression analysis, and design and verify the field programmable gate array (FPGA) framework to implement SCN model for the first time. In addition, in order to improve the performance of the SCN model based on the FPGA, the implementation of the nonlinear activation function on the FPGA is optimized, which effectively improves the prediction accuracy while considering the utilization rate of hardware resources. Experimental results based on the simulation data set and the real data set prove that the proposed FPGA framework successfully implements the SCN regression prediction model, and the improved SCN model has higher accuracy and a more stable performance. Compared with the extreme learning machine (ELM), the prediction performance of the proposed SCN implementation model based on the FPGA for the simulation data set and the real data set is improved by 56.37% and 17.35%, respectively.

## 1. Introduction

In the past few decades, the gradient-based learning method has been widely used in training neural networks, such as the backpropagation (BP) algorithm, which uses the back propagation of error to adjust the weight of the network. However, due to the improper learning step size, the convergence speed of the algorithm is very slow, and it is easy to produce a local minimum value. So, a lot of iterations are often needed to get more satisfactory accuracy. These problems have become the main bottleneck restricting its development in the application field. Therefore, improving the learning ability and generalization performance of neural network models is a challenging task. One solution is to find an appropriate architecture for the neural network model. Artificial neural networks have two important hyper-parameters, which are used to control the scale of the network: the number of layers and the number of nodes in each hidden layer. The values of these parameters must be specifically determined when configuring the network. However, there is no rule to determine the scale of the network. In the regression prediction application, most researchers set up a series of models with different scales during the training process, and then selected the best one according to the test results [1,2,3]. However, this kind of method increases the training cost and time. Thus, how to determine the ideal number of hidden layer nodes before network training is an urgent problem to be solved [4,5]. In order to solve this problem, a newly developed randomized learner model, termed stochastic configuration networks (SCNs), was proposed by Wang et al. [6]. As a single-layer feedforward neural network, the SCN belongs to the random neural network class. Although the parameters of the SCN are also randomly generated, it is different from the existing randomized learning algorithms for single layer feed-forward neural networks (SLFNNs); the SCN mainly randomly assigns the input weights and biases of hidden nodes in the light of a supervisory mechanism, and the output weights are analytically evaluated in either a constructive or selective manner. Compared to other random neural networks, this random learning model is also different from the classical random vector functional link (RVFL) network. The SCN restricts the assignment of input weights and bias by introducing inequality constraints. Under the supervisory mechanism, SCN has a universal approximation property with the increase of the number of hidden nodes [7,8,9,10]. Instead of training a model with a fixed architecture, the construction process of the SCN starts with a small sized network and then adds hidden nodes incrementally until an acceptable tolerance is achieved, then solves a global least squares problem with the current learner model to find the output weights. Its advantages ar: the minimization of a convex cost that avoids the presence of local minima, good generalization performance, and a notable representation ability [6]. Compared with deep neural networks, the SCN has lower training complexity and faster learning speed.

Nowadays, random neural networks have left impressive performance in the fields of deep learning and cognitive science. Compared with being applied to computer platforms, the implementation of random neural networks on reconfigurable digital platforms, such as field programmable gate array (FPGA), shows its huge and unique advantages: First, in the neural domain where parallelism and distributed computing are inherently involved, FPGAs have increased their speed with their very high computing power [11]. Second, with the miniaturization of component manufacturing technology [12], neural networks are becoming more and more widespread in embedded applications. Third, compared to computers, hardware systems can reduce costs by decreasing power requirements and lowering the number of components [13]. Fourth, parallel and distributed architectures have a high fault tolerance rate for system components [14], and provide support for applications that require security. Also today, a large number of mobile devices are connected to the internet, and cloud computing data centers are under excessive load. The implementation of neural networks in software requires a lot of computing resources. In order to reduce the Internet load, the collaborative use of edge and cloud computing is particularly important. FPGA-based random neural networks stand at the edge-computing perspective and move part of the computational power to data collection sources, thereby reducing the network load [15]. With the surge in data volume and the constant demand for computing power, the original computing framework consisting solely of CPUs has been unable to meet the real-time requirements of the edge-computing system, while one of the greatest value of FPGAs is that they are reconfigurable, so designs can be updated at any time, even after the hardware has been deployed in the field. By virtue of this advantage and high efficiency, FPGA is widely used in many edge computing scenarios [16]. At the same time, with the development of Internet of Things (IoT), hubs should support a large number of ultra-low power network protocols, various applications workloads, and be responsible for completing authentication, encryption, and security. This kind of changing and uncertain environment is a terrible thing for ASIC or SoC, but it is easy to implement for FPGA with very high running speed and computational efficiency. Therefore, the implementation of neural networks on FPGAs has very good development prospects and application values.

Researchers have done a lot of works on the hardware implementation of random neural networks and made many achievements. In 2012, Decherchi et al. implemented the classification prediction model of extreme learning machine (ELM) [17,18,19] on the FPGA and achieved high-precision prediction results [20]. In 2018, Ragusa et al. improved the hardware implementation model of the ELM classifier for resource-constrained devices, effectively balancing accuracy, and network complexity, and reducing resource utilization [21]. In 2019, Safaei et al. proposed a specialized system on chip (SoC) hardware implementation and design approach for embedded online sequential ELM (OS-ELM) classification, which has been optimized for efficiency in real-time applications [22].

Inspired by the above papers, this paper designs and completes the implementation of the SCN regression prediction model on the FPGA. The main contributions of this paper are listed below. (1) The SCN model exhibiting good performance in learning and generalization is investigated for regression prediction; this is the first time the SCN model on the FPGA has been implemented. (2) A new nonlinear activation function is proposed to optimize the FPGA implementation of the SCN model; this new activation function, unlike the previous ones, further considers the prediction accuracy and hardware resource utilization. (3) Experimental results from simulation and real data sets indicate that the proposed FPGA framework successfully implements the SCN regression prediction model. (4) The prediction performance of the proposed FPGA implementation of the SCN model is significantly improved compared with the same case studies for other implementation in the literature [20].

The rest of this paper is organized as follows. Section 2 describes the specifics of the SCN. Section 3 proposes the hardware architecture of the SCN. Section 4 proposes methods for improving and optimizing the performance of FPGA models. Section 5 verifies the designed SCN hardware prediction model on the simulation data set and the real industrial data set. Finally, the conclusion is drawn in Section 6.

## 2. Stochastic Configuration Networks

For a target function $f:R^{d}\to R^{m}$, suppose that an SCN model has already been built with L−1 hidden nodes, i.e., $f_{L-1}=\overset{L-1}{\underset{l=1}{\sum }}\beta_{l}\phi_{l}\left(\omega_{l}^{T}x+b_{l}\right)\;\left(L=1,2,\ldots ;f_{0}=0\right)$, where $\beta_{l}={\left[\beta_{l,1},\beta_{l,2},\ldots ,\beta_{l,m}\right]}^{T}$, and $\phi_{l}\left(\omega_{l}^{T}+b_{l}\right)$ is an activation function of the lth hidden node with random input weights $\omega_{l}$ and bias $b_{l}$. $e_{L-1}^{*}=f-f_{L-1}=\left[e_{L-1,1}^{*},\ldots ,e_{L-1,m}^{*}\right]$ denotes the residual error where $\left[\beta_{1}^{*},\beta_{2}^{*},\ldots ,\beta_{L-1}^{*}\right]=argmin_{\beta }\|f-\overset{L-1}{\underset{l=1}{\sum }}\beta_{l}\phi_{l}\|$.

Given a training data set with N sample pairs $\left\{\left(x_{n},y_{n}\right),n=1,2,\ldots ,N\right\}$, where $x_{n}\in R^{d}$ and $y_{n}\in R^{m}$, let $X\in R^{N\times d}$ and $Y\in R^{N\times m}$ represent the input and output data matrix; $e_{L-1}\left(X\right)\in R^{N\times m}$ be the residual error matrix, where each column $e_{L-1,q}\left(X\right)={\left[e_{L-1,q}\left(x_{1}\right),\ldots ,e_{L-1,q}\left(x_{N}\right)\right]}^{T}\in R^{N}$, q=1,2,…,m. Denote the output vector of the Lth hidden node $\phi_{L}$ for the input X by

(1) $$h_{L}\left(X\right)={\left[\phi_{L}\left(\omega_{L}^{T}x_{1}+b_{L}\right),\ldots ,\phi_{L}\left(\omega_{L}^{T}x_{N}+b_{L}\right)\right]}^{T}.$$

Thus, the hidden layer output matrix of $f_{L}$ can be expressed as $H_{L}=\left[h_{1},h_{2},\ldots ,h_{L}\right]$. Denoted by $\xi_{L,q}=\frac{{\left(e_{L-1,q}^{T}\left(X\right)\cdot h_{L}\left(X\right)\right)}^{2}}{h_{L}^{T}\left(X\right)\cdot h_{L}\left(X\right)}-\left(1-r-\mu_{L}\right)e_{L-1,q}^{T}\left(X\right)e_{L-1,q}\left(X\right)$,

(2) q=1,2,…,m,

where 0<r<1 and $\left\{\mu_{L}\right\}$ is a nonnegative real number sequence with $lim_{L\to +\infty }\mu_{L}=0$ subjected to $\mu_{L}\leq \left(1-r\right)$. The SCN algorithm firstly generates a large pool of $T_{\max}$ candidate nodes, namely $\left\{\phi_{L}^{\left(1\right)},\phi_{L}^{\left(2\right)},\ldots ,\phi_{L}^{\left(T_{\max}\right)}\right\}$, in varying intervals. Then, it picks up those candidate nodes whose minimal value of the set $\left\{\xi_{L,1},\ldots ,\xi_{L,m}\right\}$ is positive. Then, the candidate note $\phi_{L}^{*}$ with the largest value of $\xi_{L}=\overset{m}{\underset{q=1}{\sum }}\xi_{L,q}$ will be assigned as the *L*th hidden node for $f_{L}$. Thus, the output weight matrix of the SCN model, $\beta ={\left[\beta_{1},\beta_{2},\ldots ,\beta_{L}\right]}^{T}\in R^{L\times m}$, could be computed by the standard least squares method, that is,

(3) $$\beta^{*}=arg\underset{\beta }{min}{\left\|H_{L}\beta -Y\right\|}_{F}^{2}=H_{L}^{†}Y,$$

where $H_{L}^{†}$ is the Moore-Penrose generalized inverse of the matrix $H_{L}$, and ${\left\|\cdot \right\|}_{F}$ represents the Frobenius norm [23,24,25].

The construction process of the SCN starts with a small sized network, then incrementally adds hidden nodes followed by computing the output weights. This process continues until the model meets some termination criteria. The supervisory mechanism of the SCN guarantees the universal approximation property.

## 3. FPGA-Based Implementation of the SCN

The implementation of the SCN on the FPGA needs to balance accuracy, speed, and resource utilization. The proposed architecture should make full use of the advantages of FPGA parallel processing. Due to the fact that the SCN adopts the method of gradually increasing hidden nodes to find the optimal solution, the flexibility of the model must be fully considered in the design, so that it can make specific changes for different problems.

**Figure 1:**
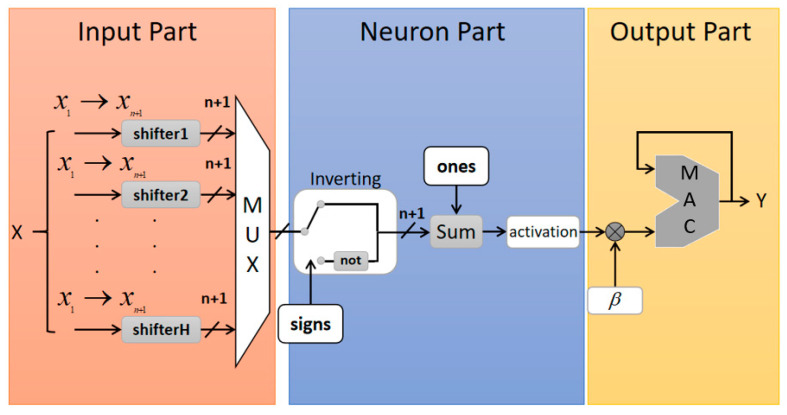
The architecture of FPGA-based SCN.

Figure 1 shows the overall architecture of the SCN inference prediction model based on the FPGA. The whole architecture includes three parts: the first part belongs to a parallel processing structure, and the second and third parts adopt a pipeline structure. The first part, Input Part, stores the feature vector $x=\left[x_{1},\ldots ,x_{n}\right]$, the weights $\omega_{j}\left(j=1,\ldots ,H\right)$ connecting the input layer to the hidden node and the bias term $b_{j}\left(j=1,\ldots ,H\right)$. The feature vector *x* adopts a signed, two-complement fixed-point representation. The binary number length of each feature vector is a+b+1, where a is the number of digits representing a positive number, b is the number of digits representing a decimal, and the remaining one is used to represent a sign bit. Negative numbers are coded by inverting their absolute value and adding 1. In order to facilitate the calculation by FPGA, the weight value $\omega_{j}$ is specially processed and can be expressed as

(4) $$\omega_{j}=sign\left(r_{1}\right)2^{-r_{2}},$$

where $r_{1}\in \left[-1,1\right]$ and $r_{2}\in \left[0,R\right]$ are random quantities (in the program, $r_{2}$ can take the following values: 1, 2, 3). If x is extended to $x=\left[x_{1},\ldots ,x_{n},1\right]$ and $\omega_{j}$ to $\omega_{j}\in R^{n+1}$, the bias term $b_{j}$ can be embedded in $\omega_{j}$. The second part, Neuron Part, receives the results of the parallel processing from the first part and calculates the output of the activation function. The third part, Output Part, receives the output of the second part and calculates the output neurons through serial processing. A finite-state machine controls the entire process, ensuring that the calculations of the third part are always one clock cycle ahead from the calculations of the first and second parts.

The specific design of each part is as follows:

(1) **Input Part:** The *Input* module stores all extended feature vectors $x_{i}={\left[x_{i1},x_{i2},\ldots ,x_{i\left(n+1\right)}\right]}^{T}\in R^{n+1}$, and the *Shifter* modules stores the absolute value of extended random weights $\omega_{j}\in R^{n+1}$. Due to the special processing of $\omega_{j}$, the FPGA can input (n+1)×H results into the *Mux* module through parallel shift calculation. The *Mux* module outputs the calculation results in turn according to the finite state machine, and outputs (n+1) items each time.
(2) **Neuron Part:** First, the *Inverting* module receives the output of the first part according to the signs of the random weights $\omega_{j}\in R^{n+1}$, and applies a bitwise NOT to the result item whose corresponding random weight is negative. Then, the output (n+1) result and the corresponding item in the ones module are input to the *Sum* module for summation, where the *Ones* module compensates the difference “1” between the calculated result and the true value due to the bitwise NOT. Finally, the result of the *Sum* module is activated by the sigmoid function of the *activation* module to obtain the output $\varphi \left(\omega_{j}x+b_{j}\right)$ of the hidden layer. The *activation* module should be a hardware implementation of the activation function. This is an extremely critical step. The implementation and optimization of the activation function in FPGA are specifically introduced in Section 4.1.
(3) **Output Part:** The *Mac* module multiplies the output $h_{j}=\varphi \left(\omega_{j}x+b_{j}\right)$ of the hidden layer by the weight $\beta_{j}$ from the hidden layer to the output layer according to the control of the state machine. The calculation results are summed by an accumulator, and then the output Y of the neural network is obtained.

## 4. Improvement and Optimization of FPGA-Based Model Performance

When the FPGA implements the single hidden layer neural network prediction model, the error sources are mainly the approximation degree of the nonlinear activation function and the difference between the data and the actual floating point number due to numerical coding.

### 4.1. Proposal of Hardware-Oriented Sigmoid Function

The sigmoid function is the most commonly used nonlinear activation function. When the SCN is implemented on FPGA, the sigmoid function (Equation (5)) is selected:

(5) $$f\left(x\right)=\frac{1}{1+e^{-x}}.$$

Because factors such as accuracy and resources must be considered at the same time, the implementation of nonlinear functions on the FPGA is very complicated [26,27]. Division and exponential operations are extremely demanding operations, requiring a large amount of area resources and slow convergence, so it is not feasible to directly implement the sigmoid function on the FPGA. Polynomial approximation and Look-Up-Table (LUT) are two common methods when the accuracy and speed meet the requirements.

In terms of the approximation of the sigmoid function, researchers have made many explorations. Tommiska proposed the piecewise linear approximation of the sigmoid function in [28], which is also the traditional method of sigmoid function implementation in hardware:

(6) $$f\left(x\right)=\{\begin{array}{cc} 0, & x<-2\\ 0.25x+0.5, & -2\leq x\leq 2\\ 1, & x>2 \end{array}.$$

**Figure 2:**
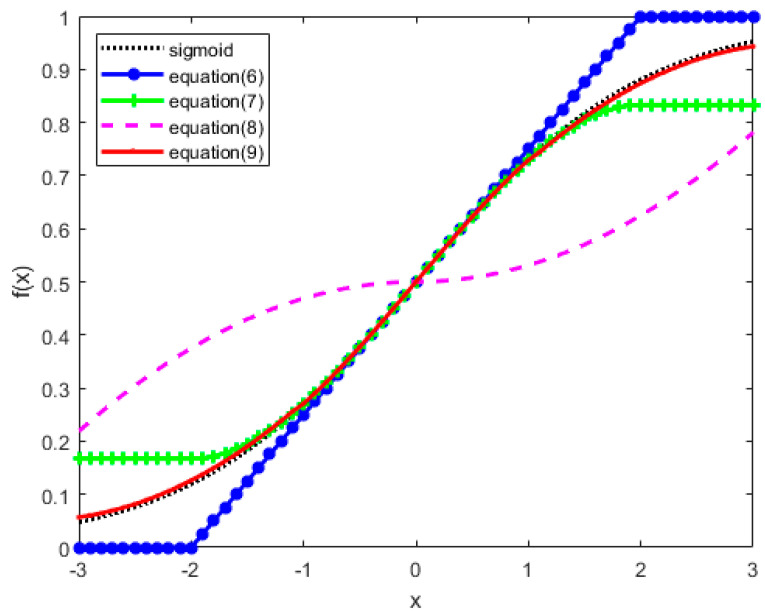
Comparison of different sigmoid functions.

This scheme was proved to be effective in solving hardware implementation of classification problems in [20]. The approximation of the activation function has little effect on classification problems, and the results of classification problems are often determined through comparison operations. The regression problem needs to directly deal with the calculation results of the FPGA-based model, which requires that the implementation of the activation function in hardware has good similarity with the original sigmoid function. It can be seen from Equation (6) that when −2≤x≤2, f(x)=0.25x+0.5 is the first-order Taylor expansion of the sigmoid function. Figure 2 shows that Equation (6) has an ideal approximation to the sigmoid function on x∈[−1,1], but not on x∈[−3,−1)∪(1,3]. Equation (7) gives the third-order Taylor expansion of the sigmoid function:

(7) $$f\left(x\right)=\{\begin{array}{cc} 0.167, & x<-2\\ -0.021x^{3}+0.25x+0.5, & -2\leq x\leq 2\\ 0.833, & 2<x \end{array}.$$

However, it is known from Figure 2 that Equation (7) does not improve the problem of Equation (6), and the approximation effect is still not ideal. Considering the use of resources, the higher-order Taylor expansion (x≥5) the sigmoid function is no longer suitable for hardware implementation. Therefore, a combinational approximation should be found on the basis of Equation (7). A method of Piecewise second-order approximation of sigmoid function was proposed in [29] (Equation (8)):

(8) $$f\left(x\right)=\{\begin{array}{cc} -0.03125x^{2}+0.5, & -3\leq x<0\\ 0.03125x^{2}+0.5, & 0\leq x\leq 3 \end{array}.$$

Due to excessive consideration of resource utilization in [29], the proposed results are severely impaired in the approximation of the sigmoid function shown in Figure 2. The Equation (9) proposed in this paper improves the second-order approximation of Equation (8) on the basis of Equation (7), and applies it to x∈[−3,−1)∪(1,3]:

(9) $$f\left(x\right)=\{\begin{array}{cc} 0.03913x^{2}+0.2651x+0.5, & -3\leq x<-1\\ -0.021x^{3}+0.25x+0.5, & -1\leq x\leq 1\\ -0.03913x^{2}+0.2651x+0.5, & 1<x\leq 3 \end{array}.$$

As shown in Figure 2, comparing with the sigmoid function, Equation (9) almost perfectly presents the sigmoid function.

In fact, many researchers have given different approximation methods for the implementation of the sigmoid function on FPGA. In 2012, Panicker et al. proposed a piecewise linear approximation method in [30]:

(10) $$f\left(x\right)=\{\begin{array}{cc} 0.03125\left|x\right|+0.84375, & 2.375\leq \left|x\right|\leq 3\\ 0.125\left|x\right|+0.625, & 1\leq \left|x\right|<2.375\\ 0.25\left|x\right|+0.5, & 0\leq \left|x\right|<1 \end{array}.$$

In 2015, Khodja et al. proposed a piecewise second-order approximation method in [31]:

(11) $$f\left(x\right)=\{\begin{array}{cc} 0.0332x^{2}+0.2549x+0.5, & -3\leq x<0\\ -0.0332x^{2}+0.2549x+0.5, & 0\leq x\leq 3 \end{array}.$$

In 2016, Ngah et al. also proposed a piecewise second-order approximation method in [32]:

(12) $$f\left(x\right)=\{\begin{array}{cc} 0.5\times {\left(1-0.25\left|x\right|\right)}^{2}, & -3\leq x<0\\ 1-0.5\times {\left(1-0.25\left|x\right|\right)}^{2}, & 0\leq x\leq 3 \end{array}.$$

In order to qualitatively analyze the approximation of sigmoid function by Equations (6)–(12), according to the method of [29], the maximum and average errors are used to evaluate the approximation degree of the sigmoid function. If a function f(x) is approximated by a function $\hat{f}\left(x\right)$ the interval $x\in \left(\eta_{0},\eta_{1}\right)$, the average and maximum errors are obtained by uniformly sampling x on $10^{6}$ equally spaced points in the domain of $\left(\eta_{0},\eta_{1}\right)$.

(13) $$\{\begin{array}{c} Average\;Error=\frac{\sum_{i=0}^{10^{6}-1}\left|\hat{f}\left(x_{i}\right)-f\left(x_{i}\right)\right|}{10^{6}}\\ Maximum\;Error=\underset{\eta_{0}<x_{i}<\eta_{1}}{\max}\left|\hat{f}\left(x_{i}\right)-f\left(x_{i}\right)\right| \end{array}$$

According to Equation (13), when $\eta_{0}=-3,\;\eta_{1}=3$, the average and maximum errors corresponding to Equations (6)–(12) are shown in Table 1.

As shown in Table 1, the average error between the Equation (9) proposed in this paper and the sigmoid function is less than 0.001, which best completes the approximation of the sigmoid function.

Table 2 lists the resource utilization rate (Proportion of Slice LUTS used to available Slice LUTS) of Equations (6), (7) and (9) after synthesizing on Xilinx’s FPGA XC7Z020CLG400-2. As can be seen from Table 2, the resource utilization rate of Equation (9) is similar to that of Equations (6) and (7). On the regression model that requires higher calculation accuracy, the proposed Equation (9) can make the calculation result of FPGA prediction model closer to the real value, which is a better choice for balancing accuracy and resource utilization.

### 4.2. Format of Numerical Representation on FPGA

The FPGA architecture uses the signed, two-complement fixed-point representation for numbers. The difference between the encoded number and the target value may also be one of the reasons for the error. Table 2 shows the comparison between the binary number and the target value in the 16-bit (a=4,b=11) or 21-bit (a=4,b=16) encoding mode.

Table 3 shows that the 16-bit and 21-bit encoded numbers are almost the same as the target values, proving that the results of the two different encoded numbers are basically equal. In order to save resources, the FPGA-based model uses a 16-bit coding format.

## 5. Experimental Results

The regression prediction model based on the FPGA is tested on simulation data set and real industrial data set. The simulation data set consists of 1200 patterns (located in 1-eigenvalue space), of which the training set contains 600 patterns and the test set contains 600 patterns. The real hot-rolled strip crown data set were collected from a 1780 mm hot strip production line of a company in Hebei Province, China. In a hot-rolled process, crown is defined as the difference of thickness between the center and a point 40 mm from the edge of the strip. For strip products, smaller crown is required, which can save materials and reduce costs. Therefore, control of strip crown is a high priority for hot-rolled production process. The crown of the strip is decided by the 3D deformation in finishing mill, it can be regarded as the reflection of the cross-sectional shape of roll gap at the outlet of finishing mill. Thus, all the factors which can affect the cross-sectional shape of roll gap at the outlet of deformation zone are the factors that affect the crown value of strip. In this paper, nine important attributes in hot-rolled strip production are selected as input variables. They are: Cooling water flow of rolling mill (%), Entrance temperature (°C), Exit temperature (°C), Strip width (m), Entrance thickness (mm), Exit thickness (mm), Bending force (kN), Rolling force (kN) and Entry profile (μm). The real hot-rolled strip crown data set contains 474 patterns (located in 9 eigenvalue space), of which the training set contains 380 patterns and the test set contains 94 patterns. All data are normalized within the range of [−1, 1]. The experimental test realizes the regression prediction of the SCN based on the FPGA. In [20], the ELM model implemented on the FPGA is applied to the detection of classification problems. We modified the FPGA-based ELM model in [20] and adopted it to the regression problem. We give the experimental results of the FPGA-based ELM model on the simulation data set and the real data set, and compare it with the proposed FPGA-based SCN model to explain the superiority of the SCN. The FPGA used in the experiment is Xilinx’s FPGA XC7Z020CLG400-2.

**Figure 3:**
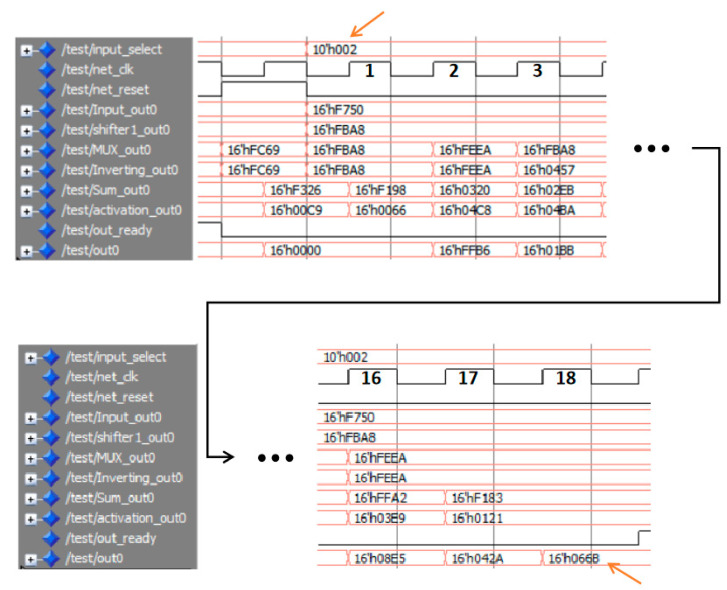
Functional simulation of the field programmable gate array (FPGA)-based implementation of the stochastic configuration network (SCN).

Figure 3 shows the functional simulation results of the input and output signals of the modules in the FPGA architecture, including: clock signal (net_clk), reset signal (net_reset), pattern selection signal (input_select), an output signal of the *Input* module (Input_out0—an eigenvalue), an output of the *Shifter* module (Shifter1_out0), an output of the *Mux* module (Mux_out0), an output of the *Inverting* module (Inverting_out0), an output of the *Sum* module (Sum_out0), an output of the *activation* module (activation_out0), output enable signal (out0_ready) of the *Mac* module which indicates that the result has been calculated and the final output of the system (out0) in the *Mac* module. Taking the signal in Figure 3 as an example, it represents the calculation process of the data of one pattern in the FPGA, where the value “10′h002” of the Signal *input_select* represents the calculation of the second pattern currently being performed. The calculation process in the FPGA given in Figure 3 has 18 clock cycles of the Signal *net_clk*, corresponding to the 18 hidden nodes in the SCN model. The Signal *out_ready* in the figure is the flag signal for calculating the data of each pattern. The Signal *out_ready* is set low at the rising edge of the Signal *net_reset*, indicating that this pattern data calculation process starts, and the low level state is always maintained during the calculation process. Then after the calculation is completed (that is to say, after the 18 clock cycles of the Signal *net_clk*), the Signal *out_ready* is set high, which indicates that the pattern data calculation process ends. After the rising edge of the Signal *out_ready* appears, the value “16’h066B” of the Signal *out0* is the prediction result of the second pattern. The specific implementation process of the SCN on the FPGA is as follows: After being triggered by the reset signal (*net_reset*), the *Shifter* module acquires an input vector $\left[x_{1},x_{2},\ldots ,x_{N}\right]$ from the *Input* module. At the rising edge of the next clock, the *Mux* module receives the result [sh1,sh2,…,shN] from the *Shifter* module in parallel. The *Inverting* module processes the data and outputs it according to the signs of the input weights. After another rising edge of the clock, the *Sum* module adds up the data and activates the data through the *activation* module. The *Mac* module accumulates the activated data after another rising edge of the clock. When all data accumulation is completed, the Signal out0_ready generates a rising edge, and then the final output of the system can be read out from the signal out0.

**Figure 4:**
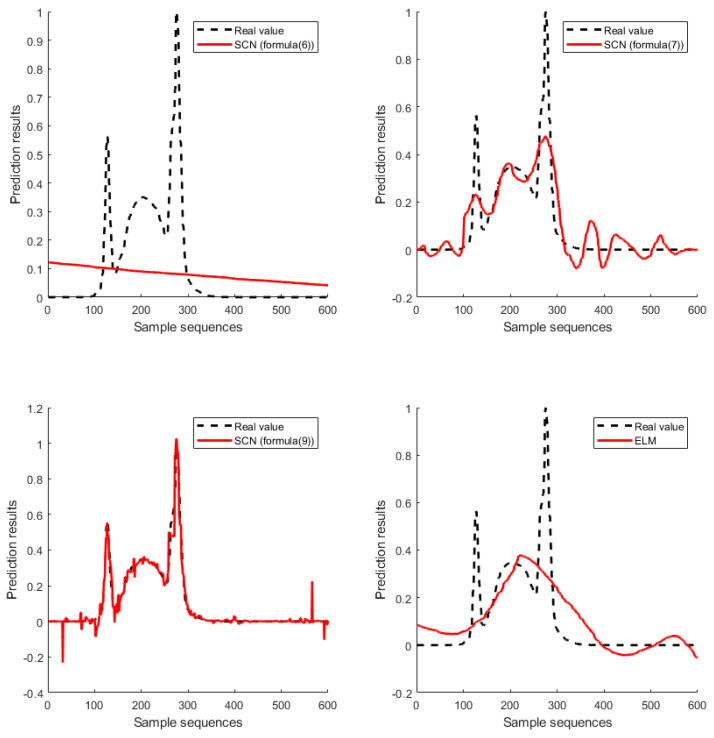
Comparison of SCN (Formulas (6), (7), (9)) and ELM implementation on the simulation data set.

**Figure 5:**
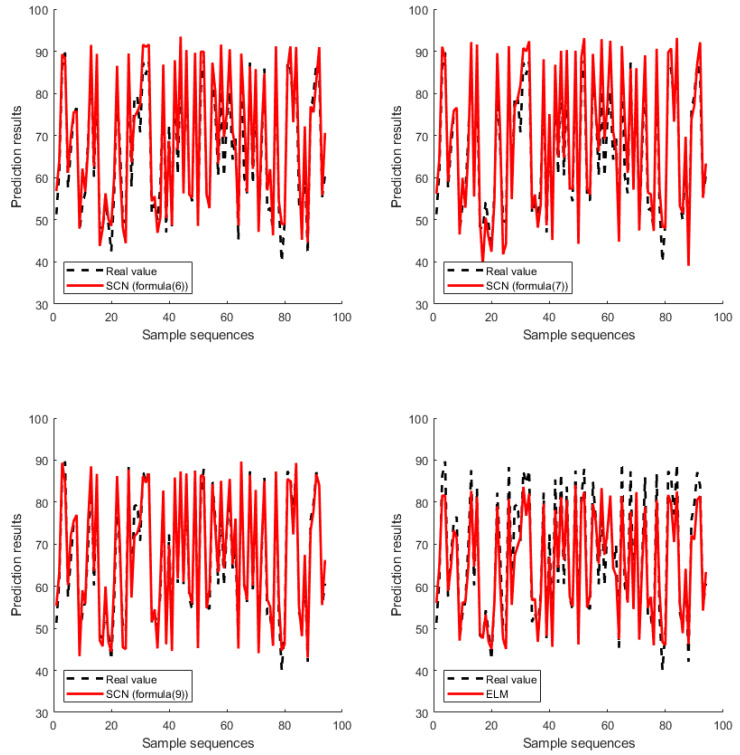
Comparison of SCN (Formulas (6), (7), (9)) and ELM implementation on the real data set.

Figure 4 and Figure 5 show the results of FPGA regression prediction model on the simulation data set and real data set. In the simulation data set, the number of hidden layer nodes of the SCN and ELM were 18 and 35 respectively, while in the real data set, the number of hidden layer nodes of the SCN and the ELM were 42 and 55 respectively. As can be seen from Figure 4, for the simulation data set, the SCN model based on Equation (7) and the ELM model can only predict the general trend of the real value, while the SCN model based on Equation (6) cannot predict the trend of the real value, and only the SCN model based on Equation (9) can predict the real value well. As can also be seen from Figure 5, for the real data set, the SCN model based on Equation (9) has the best prediction result, and the predicted value almost completely coincides with the real value, while the prediction result of other models is relatively poor. Therefore, both the simulation data set and real data set prove that the FPGA implementation of the SCN model based on Equation (9) proposed in this paper has the best prediction performance.

In order to quantitatively analyze the implementation effect of the optimized SCN on the FPGA, Table 4 shows the average values of 30 groups of root-mean-square errors (RMSE) of the implementation results of FPGA-based SCNs and ELM, computer-based SCN and ELM on two data sets. Considering that FPGA is limited by hardware resources and calculation accuracy, and its calculation capability is relatively weak compared with that of a computer, Table 4 takes the prediction results of computer as benchmark, and compares the implementation accuracy of the proposed model on FPGA with that of a computer. As can be seen from Table 4, in the simulated data set, the implementation result of the SCN on the computer is worse than that of the ELM, but in the real data set, the implementation result of the SCN on the computer is better than that of the ELM. Therefore, the advantages of the SCN based on computer implementation are not obvious. Compared with the implementation of the SCN on computer, the prediction accuracy of the SCN implemented on FPGA with Equations (6) and (7) is greatly reduced. Using the sigmoid function proposed in Equation (9), the prediction accuracy of the SCN implemented on FPGA is obviously the best, which is completely better than the ELM, especially in real data set, and is almost the same as the implementation accuracy of the SCN on the computer. The results fully demonstrate the strong generalization ability and high prediction accuracy of the FPGA-based the SCN proposed in this paper.

Table 5 and Table 6 show the resource utilization and power consumption of SCNs and ELM implemented on the FPGA. The analysis of Table 5 and Table 6 shows that, whether it is the simulation data set or the real data set, compared with the ELM, the resource utilization and power consumption of SCN implemented on FPGA is lower. However, the resource utilization and power consumption of SCNs based on different equations are basically the same. In order to analyze the running speed of the SCN model based on the FPGA, Table 7 shows the actual clock frequencies of the SCN and the ELM implemented on the two data sets for comparison. As can be seen from Table 7, the experiments on both data sets prove that the SCN implemented on FPGA runs faster than ELM. The fundamental reason is that the SCN needs fewer hidden layer nodes to reach the optimal solution, therefore, FPGA-based implementation of the SCN has lower resource utilization and power consumption and faster operation speed than the ELM.

## 6. Conclusions

This paper adopts the SCN to regression analysis, and verifies the FPGA framework to implement the SCN model. Based on the balance between prediction accuracy and resource utilization, this paper cuts in from multiple perspectives to improve the performance of the SCN model based on the FPGA. The implementation of the activation function on the FPGA is optimized, which effectively improves the prediction accuracy while considering the utilization rate of hardware resources. Experimental results based on simulation and real data sets prove that the proposed FPGA framework successfully implements the SCN regression prediction model, and the improved SCN model has higher accuracy and more stable performance. Compared with other implementations in the literature [20], the prediction performance of the proposed the SCN implementation model based on the FPGA for simulation data set and real data set is improved by 56.37% and 17.35% respectively. In addition to the prediction accuracy, this paper also compares and analyzes the experimental results from the aspects of resource utilization rate, power consumption and running speed. The experimental results fully demonstrate the performance advantages of the FPGA-based SCN implementation architecture proposed in this paper. Some studies have shown that the SCN model is not only used for regression prediction but also can be used for recognition and detection. In the future, we can extend the FPGA-based SCN prediction model to more complex scenes. The use of FPGA-based SCN model will be of great significance for edge computing and alleviating the computing pressure of the cloud computing center.

## Figures and Tables

**Table 1:** The average and maximum errors of Equations (6)–(12).

	Average Error	Maximum Error
Equation (6)	0.048187	0.1192
Equation (7)	0.035147	0.11924
Equation (8)	0.1914	0.25718
Equation (9)	0.000606	0.00244
Equation (10)	0.006037	0.018941
Equation (11)	0.006228	0.013326
Equation (12)	0.008038	0.016176

**Table 2:** Resource utilization of the sigmoid function with different approximation methods.

	Equation (6)	Equation (7)	Equation (9)
Resource utilization	0.0282%	0.0320%	0.0469%

**Table 3:** Comparison of encoded binary numbers with target values.

$\mathrm{Target}\;\mathrm{Value}:\;\lambda_{target}$	Binary Number	$\mathrm{Decimal}\;\mathrm{Value}\;\mathrm{for}\;\mathrm{Binary}\;\mathrm{Number}:\;\lambda_{decimal}$	$\mathrm{Relative}\;\mathrm{Error}:\;\frac{\left|\lambda_{target}-\lambda_{decimal}\right|}{\left|\lambda_{target}\right|}$
0.294294	0000001001011010	0.293945	0.119%
−1.086123	1111011101010000	−1.085937	0.017%
0.294294	000000100101101010110	0.294281	0.004%
−1.086123	111101110100111110100	−1.086120	0.003%

**Table 4:** Average root-mean-square error (RMSE) of the prediction results.

	Simulation Data Set	Real Data Set
FPGA-based SCN (Equation (6))	0.1625	4.9169 µm
FPGA-based SCN (Equation (7))	0.1056	4.7567 µm
FPGA-based SCN (Equation (9))	0.0551	3.5783 µm
FPGA-based SCN (Equation (10))	0.0614	3.7821 µm
FPGA-based SCN (Equation (11))	0.0643	3.7994 µm
FPGA-based SCN (Equation (12))	0.0626	3.7187 µm
FPGA-based ELM	0.1263	4.3296 µm
Computer-based SCN	0.0150	3.5404 µm
Computer-based ELM	0.0126	4.2290 µm

**Table 5:** Resource utilization of FPGA-based prediction models.

	SCN (Equation (6))	SCN (Equation (7))	SCN (Equation (9))	ELM
Simulation data set	2.29%	2.30%	2.32%	2.48%
Real data set	1.65%	1.66%	1.68%	2.14%

**Table 6:** Power consumption of FPGA-based prediction models.

	SCN (Equation (6))	SCN (Equation (7))	SCN (Equation (9))	ELM
Simulation data set	0.991 W	0.991 W	0.991 W	0.993 W
Real data set	1.039 W	1.039 W	1.039 W	1.043 W

**Table 7:** Actual clock frequency of FPGA-based prediction models.

	SCN (Equation (9))	ELM
Simulation data set	43.1 MHz	31.7 MHz
Real data set	48.6 MHz	41.8 MHz
